# Impact of Somatic Gene Mutations on Prognosis Prediction in De Novo AML: Unraveling Insights from a Systematic Review and Meta-Analysis

**DOI:** 10.3390/cancers17193189

**Published:** 2025-09-30

**Authors:** Amal Elfatih, Nisar Ahmed, Luma Srour, Idris Mohammed, William Villiers, Tara Al-Barazenji, Hamdi Mbarek, Susanna El Akiki, Puthen Veettil Jithesh, Mohammed Muneer, Shehab Fareed, Borbala Mifsud

**Affiliations:** 1 Genomics and Precision Medicine, College of Health and Life Science, Hamad Bin Khalifa University, Doha 34110, Qatar; anisar@wustl.edu (N.A.); lusr30193@hbku.edu.qa (L.S.); imohammed-c@sidra.org (I.M.); wvilliers@hbku.edu.qa (W.V.); jveettil@hbku.edu.qa (P.V.J.); 2 Qatar Precision Health Institute, Doha 5825, Qatar; hmbarek@qf.org.qa; 3 Department of Medical and Molecular Genetics, King’s College London, London WC2R 2LS, UK; 4 Department of Biomedical Sciences, College of Health Sciences, Qatar University, Doha 2713, Qatar; ta1706200@student.qu.edu.qa (T.A.-B.); selakiki@hamad.qa (S.E.A.); 5 Diagnostic Genomic Division, Department of Lab Medicine & Path, Hamad Medical Corporation, Doha 3050, Qatar; 6 Weill Cornell Medicine—Qatar, Qatar Foundation—Education City, Doha 24144, Qatar; 7 Department of Plastic Surgery, Hamad Medical Corporation, Doha 3050, Qatar; mmyousif78@yahoo.com; 8 Hematology Department, NCCCR, Hamad Medical Corporation, Doha 3050, Qatar; shehabfareed@yahoo.com; 9 William Harvey Research Institute, Queen Mary University London, London WC2A 3JB, UK

**Keywords:** acute myeloid leukemia, somatic mutation, overall survival, relapse free survival

## Abstract

**Background:** Wide application of genome sequencing technologies has highlighted extensive genetic diversity in Acute Myeloid Leukemia (AML), yet the specific roles of individual genes remain unclear. This systematic review and meta-analysis aims to provide robust evidence for the prognostic impact of somatic gene mutations in de novo AML patients, while also exploring the prevalence of these mutations. **Methods:** Eligible studies were identified from PubMed and Scopus, with a focus on those reporting the prognostic influence of somatic gene mutations on overall survival (OS) or relapse-free survival (RFS) when compared to wild-type carriers. We calculated the pooled prevalence with 95% confidence intervals to assess the frequency of these mutations, and the pooled Hazard Ratio (HR) to compare OS and RFS associated with specific gene mutations. **Results:** We evaluated 53 somatic gene mutations using 80 studies, involving 20,048 de novo AML patients. The analysis revealed that the most prevalent affected genes were NPM1 (27%), DNMT3A (26%), and FLT3-ITD (24%). Mutations in CSF3R, TET2, and TP53 were significantly associated with poorer OS or RFS (*p* < 0.05). Sensitivity analysis confirmed that ASXL1, DNMT3A, and RUNX1 mutations were consistently linked to inferior OS or RFS. In contrast, *CEBPAdm* mutations were associated with favorable OS [HR = 0.39 (0.30–0.50)] and RFS [HR = 0.44 (0.37–0.54)]. Subgroup analysis showed that FLT3-ITD mutations were consistently associated with worse OS or RFS across all subgroups, though no significant subgroup differences were noted. No significant impact on OS or RFS was observed for mutations in GATA2, FLT3-TKD, KRAS, NRAS, IDH1, and IDH2. **Conclusions:** These findings provide critical insights into AML prognosis, aiding clinical decision-making and improving risk stratification strategies.

## Simple Summary

This systematic review and meta-analysis aimed to evaluate the prevalence and prognostic impact of somatic gene mutations in de novo Acute Myeloid Leukemia (AML) patients. Data from 80 studies involving 20,048 patients and 53 somatic mutations were analyzed. The most frequent mutation was NPM1 (27%). Mutations in CSF3R, TET2, TP53, ASXL1, DNMT3A, and RUNX1 were associated with worse overall survival (OS) and relapse-free survival (RFS), while CEBPA biallelic mutations were linked to favorable outcomes. FLT3-ITD mutations showed a consistently poor prognostic impact across all subgroups. No significant associations with OS or RFS were found for GATA2, FLT3-TKD, KRAS, NRAS, IDH1, and IDH2. The results of this study enhance the understanding of the genetic landscape of AML and support improved risk stratification and clinical decision-making.

## 1. Introduction

The introduction of next-generation sequencing (NGS) has provided novel insights into the molecular underpinnings of acute myeloid leukemia (AML). In clinical practice, molecular analysis has been integral to AML risk stratification and prognostication [1,2]. Recent advancements in treatment protocols have contributed to improving the outcome of AML, with 5-year survival rates of 40–45% among AML patients up to age 50–55 years, and 30–35% 5-year survival rates in patients aged 60 years and older [3]. Despite relatively high rates of initial response, the occurrence of chemotherapy resistance and disease relapse varies from approximately 30–35% in younger patients with favorable risk factors to 70–80% in older patients with adverse risk factors [4]. This suggests that the overall prognosis remains notably unfavorable despite the considerable progress.

Identifying recurrent somatic mutations has significantly advanced our understanding of the sophisticated biological and clinical diversity observed among AML patients. Based on etiology, three main types of AML exist: de novo AML, secondary AML, and therapy-related AML. De novo AML refers to myeloid leukemia that arises without a prior history of antecedent hematological conditions or exposure to genotoxic therapy. Although some somatic mutations overlap between these subtypes, de novo AML is enriched with a unique set of somatic mutations that can influence prognosis. These mutations, often involving critical genes associated with intracellular signaling, epigenetic modifications, transcriptional regulation of gene expression, apoptosis, and cell cycle regulation, contribute to heightened proliferation and disrupted differentiation of hematopoietic precursors [5]. In the clinical realm, recurrent somatic mutations are recognized in updated classifications published by the WHO and ICC expert groups [6] as factors influencing the initial risk stratification and crucial predictors of outcomes of de novo AML patients. Significantly, a consensus has been reached regarding the significance and practical applicability of specific somatic mutations, categorizing them into distinct prognostic groups, namely, favorable, intermediate, and adverse outcomes, as outlined in the European LeukemiaNet (ELN) 2022 report [2]. Additionally, some of the more recently identified mutations, such as IDH1/2, have been subjects of investigation as predictive factors in various studies and are gaining importance in comprehensive mutational profiling of AML [7]. Numerous studies have been conducted to evaluate the potential prognostic significance of somatic mutations in AML patients, yielding variable results. In this context, we explored the prevalence of somatic mutations in de novo AML and conducted a meta-analysis to provide robust evidence for the prognostic impact of somatic mutations in order to guide decisions regarding clinical testing of these mutations.

## 2. Methods

The protocol for this systematic literature review and meta-analysis was registered in the International Prospective Register of Systematic Reviews [8] (PROSPERO) under the ID number CRD42023405242. Furthermore, this study followed Preferred Reporting Items for Systematic Reviews and Meta-Analysis (PRISMA) recommendations [9].

### 2.1. Search Strategy

PubMed and Scopus were systematically searched up to 13 November 2024. Keywords, along with their synonyms and combinations using Boolean operators, were applied across all databases. The search terms included: “AML”, “acute myeloid leukemia”, “somatic mutations”, “driver mutations”, “AML progression”, “leukemia relapse”, “prevalence”, and “frequency.” The literature search was performed without regional limitations. All identified studies were imported into the Rayyan tool [10] to remove duplicates and further screen the literature against the eligibility criteria.

### 2.2. Eligibility Criteria

Studies were included if they met the following criteria: (1) human studies involving all age groups diagnosed with de novo AML, (2) studies reporting the prognostic impact of somatic gene mutations in AML patients compared to wild-type carriers of the same mutation, (3) studies reporting overall survival (OS) and/or relapse-free survival (RFS) rates, and (4) studies published in English. Studies were excluded if they: (1) were literature reviews, case reports, small cohorts (fewer than 10 participants), conference abstracts, or editorial letters, (2) involved cohorts with overlapping leukemia phenotypes, (3) involved non-de novo AML, (4) included mixed AML types where non-de novo cases exceeded 10% of the total cohort, or (5) provided insufficient data to assess the impact of individual somatic mutations or to calculate the hazard ratio. In cases where multiple reports were published on the same cohort, we selected the most recent and comprehensive study.

### 2.3. Data Extraction

Three researchers (A.E., N.A., and L.S.) independently screened the titles and abstracts according to the inclusion and exclusion criteria. The full texts were assessed, and the senior investigator (B.M.) was consulted to resolve any disagreements during the literature screening process. The studies ultimately included in the analysis were selected based on quality assessment. A standardized form was used to extract information from the included articles. The extracted information included the first author, year of publication, country of origin, sample size, age, male ratio, median follow-up duration, type of molecular test, tested somatic mutations, and co-occurring somatic mutations.

Survival outcome information was incorporated into this meta-analysis, including the hazard ratio (HR) for OS, defined as the time from diagnosis or study entry to the last recorded vital status, and RFS, defined as the time from diagnosis or study entry to treatment failure, relapse, death, or last follow-up. Multivariate analysis was preferred for calculating HR and 95% confidence intervals (CIs). If multivariate data were unavailable, univariate analyses or estimates derived from Kaplan–Meier survival curves were used. To estimate the HR from Kaplan–Meier survival curves, we used the method previously proposed by Tierney and colleagues [11], where the overall HR was calculated by extracting survival probabilities at specific time points, estimating the non-overlapping time interval, and then combining the ratio of estimated HR between groups for each interval to obtain an overall HR, using the spreadsheet provided in Tierney and colleague’s publication.

### 2.4. Quality Assessment

The quality of the primary manuscripts was assessed by two reviewers (M.M. and S.F.) using the Newcastle–Ottawa Quality Assessment Scale (NOS), which is designed for evaluating observational and case–control studies [12]. Only studies scoring five or higher (fair or good quality) were considered for inclusion in this study. Any discrepancies between the reviewers were addressed through discussion and resolved by consensus.

### 2.5. Statistical Analysis

Statistical analyses were performed using R software, version 4.3.2. The pooled prevalence and 95% CIs were calculated to estimate the overall prevalence of tested mutations among patients with de novo AML. Pooled HRs less than 1.00 indicated a better prognostic effect in AML patients with tested somatic gene mutations compared to those harboring the wild-type form of the respective gene, while HRs greater than 1.00 indicated a worse prognostic effect. Results were considered statistically significant if the 95% CIs did not include 1.00 and the *p*-value was less than 0.05. Given the known clinical heterogeneity among studies, the random-effects model was used for all statistical analyses. Heterogeneity among primary studies was assessed using the Q test, with a *p*-value less than 0.10 indicating statistically significant heterogeneity (*P^h^*). Additionally, heterogeneity was evaluated using the *I*^2^ statistic, with values of 25%, 50%, and 75% considered low, moderate, and high heterogeneity, respectively [13]. Genes with statistically significant prognostic effects and significant heterogeneity were further analyzed using a leave-one-out sensitivity analysis and subgroup analyses to explore potential sources of heterogeneity, including subject region, age, and data type. Publication bias was initially assessed by visual inspection of the funnel plot and further evaluated using the Egger- [14] and Begg-tests [15]. A *p*-value less than 0.05 indicated the presence of publication bias.

## 3. Results

### 3.1. Search Results

**Figure 1:**
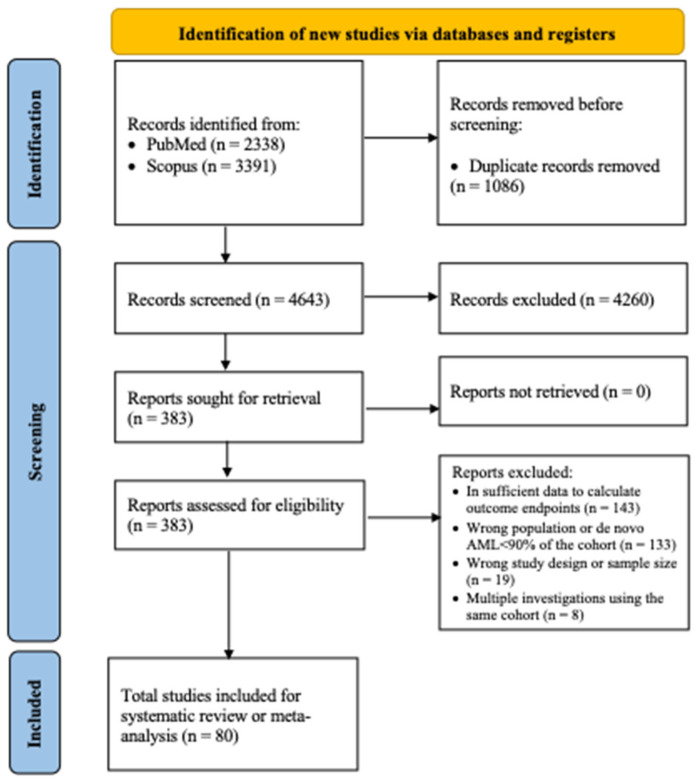
Prisma flow diagram of the study selection.

The results of the review process are summarized in the PRISMA flow diagram, which illustrates the article selection process in Figure 1. Systematic searches of potentially relevant articles published up to 13 November 2024, identified 2338 abstracts in PubMed and 3391 in Scopus. After removing 1086 duplicates, 4643 articles remained for screening against the eligibility criteria, and 383 studies proceeded to full-text review. Of these 383 studies, 143 were excluded due to insufficient data to calculate outcome endpoints, 133 had incorrect population or cohorts where >10% of cases were non-de novo, 19 had inappropriate study designs or small sample sizes, and 8 had overlapping cohorts across multiple studies. As a result, 80 publications comprised the evidence base for this review and meta-analysis. All included manuscripts were of high or fair quality according to the NOS (Table 1).

### 3.2. Studies Characteristics

The characteristics of the 80 included studies [16,17,18,19,20,21,22,23,24,25,26,27,28,29,30,31,32,33,34,35,36,37,38,39,40,41,42,43,44,45,46,47,48,49,50,51,52,53,54,55,56,57,58,59,60,61,62,63,64,65,66,67,68,69,70,71,72,73,74,75,76,77,78,79,80,81,82,83,84,85,86,87,88,89,90,91,92,93,94,95] are summarized in Table 1. These studies were conducted in over 21 countries, with the largest number originating from China (*n* = 22), Japan (*n* = 14), the US (*n* = 7), and Egypt (*n* = 7). Additionally, three studies [17,62,80] were multicenter studies involving more than two countries. The 80 studies covered a total of 20,048 de novo AML patients. Of these, 7170 patients (35.8%) were from adult-only cohorts, 2783 patients (13.9%) were pediatric, 9729 patients (48.5%) were included in mixed-age cohorts, and 366 patients (1.8%) were studied in cohorts with unknown age distribution. Males accounted for approximately 53% (10,572 patients) of the sample size. The studies were published between 2004 and 2023, with the highest number of publications in 2022 (11 studies). Various types of molecular testing were used, with the most common methods being direct sequencing (30 studies) and NGS gene panels (23 studies). A total of 53 genes were analyzed, with FLT3-ITD being the most frequently assessed for prognostic impact (32 studies), followed by NPM1, NRAS, DNMT3A, and cKIT (16 studies), and then WT1 (14 studies) and CEBPA biallelic (CEBPAdm) (13 studies).

### 3.3. Frequency of Somatic Genetic Alterations in De Novo AML Patients

**Figure 2:**
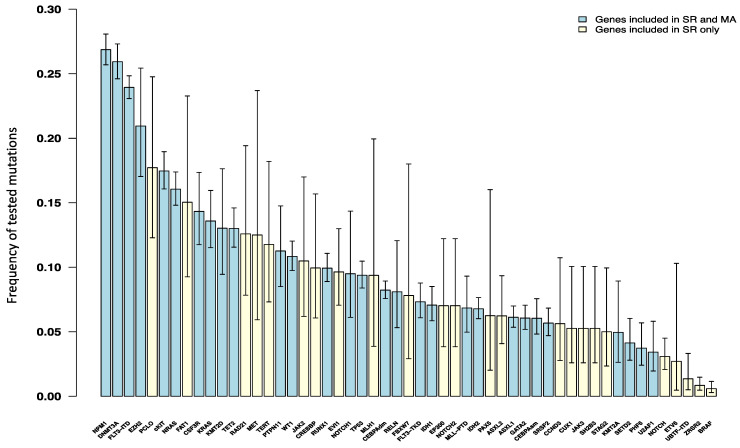
Bar plot displays the frequency distribution of somatic gene mutations among de novo AML patients. Each bar on the *x*-axis corresponds to a specific gene with a mutation, whereas the *y*-axis illustrates the frequency of these mutations across studies. Bars in yellow are genes, which were only included in the systematic review (SR), while bars in blue represent genes that were included in both systematic review and meta-analysis (MA).

We calculated the pooled prevalence and 95% CIs for the 53 genes across 80 publications to assess the overall prevalence of various mutations in patients with de novo AML. Among the most common somatic mutations in these patients were NPM1 (26.87%), DNMT3A (25.93%), and FLT3-ITD (23.95%) (Figure 2). In contrast, somatic mutations in UBTF-ITD (1.36%), ZRSR2 (0.84%), and BRAF (0.58%) were among the least prevalent of the 53 tested genes.

Additionally, we calculated the overall prevalence of somatic mutations in studies that included only pediatric cohorts (13 studies). In these studies, NRAS (191/890; 21.46%), FLT3-ITD (248/1626; 15.25%), and cKIT (68/451; 15.08%) were the most frequently mutated genes (Supplemental Figure S1).

### 3.4. Molecular Determinants of Overall Survival and Relapse-Free Survival

The prognostic impact of ASXL2, BRAF, CCND3, CREBBP, CUX1, EP300, ETV6, EVI1, FAT1, FBXW7, JAK2, JAK3, MET, MLH1, NOTCH, NOTCH2, PAX5, PCLO, RAD21, SH2B3, STAG2, TERT, UBTF-ITD, and ZRSR2 on OS or RFS in de novo AML is summarized in Supplemental Table S1. The effect of these 24 genes on OS or RFS was reported only once in the 80 publications included in our analysis, and as a result, they were not included in the meta-analysis. The remaining 29 genes were evaluated by pooling the HRs for OS or RFS for each gene.

### 3.5. Pooled Analysis of Somatic Gene Mutations with Significant Impact on OS or RFS

**Figure 3:**
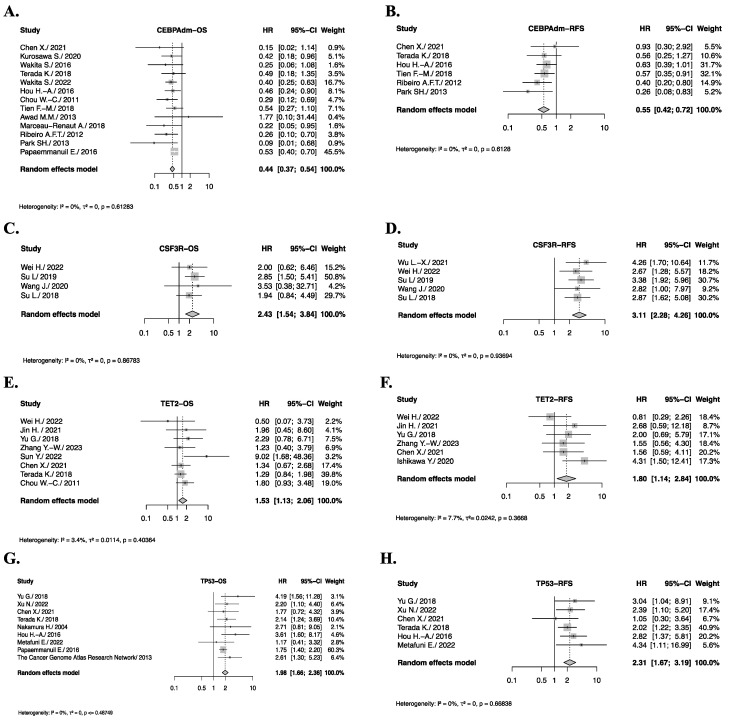
Genes with significant OS or RFS impact in de novo AML patients. Forest plot illustrating hazard ratios (HR) and their corresponding confidence intervals (CI) for overall survival (OS) or relapse free survival (RFS) in de novo AML patients. (**A**), Pooled HRs and 95% CI for CEBPA biallelic (CEBPAdm) OS. (**B**), Pooled HRs and 95% CI for CEBPAdm RFS. (**C**), Pooled HRs and 95% CI for CSF3R OS. (**D**), Pooled HRs and 95% CI for CSF3R RFS. (**E**), Pooled HRs and 95% CI for TET2 OS. (**F**), Pooled HRs and 95% CI for TET2 RFS. (**G**), Pooled HRs and 95% CI for TP53 OS. (**H**), Pooled HRs and 95% CI for TP53 RFS.

The literature search on the impact of the CEBPAdm (CEBPA biallelic) somatic gene mutations on OS and RFS in de novo AML patients identified 13 and 6 relevant studies, respectively. In the studies included in the pooled analysis, CEBPAdm mutant de novo AML patients showed statistically significant, favorable differences in both OS [HR = 0.44 (0.37–0.54), *p* < 0.0001, *I*^2^ = 0%, *P_h_* = 0.61] and RFS [HR = 0.55 (0.42–0.72), *p* < 0.0001; *I*^2^ = 0%, *P_h_* = 0.61] compared to CEBPAdm wild-type patients (Figure 3A,B). In contrast, the prognostic impact of CSF3R [HR = 2.43 (1.54–3.84), *p* = 0.0001, *I*^2^ = 0%, *P_h_* = 0.87], TET2 [HR = 1.53 (1.13–2.06), *p =* 0.0059, *I*^2^ = 3.4%, *P_h_* = 0.40], and TP53 [HR = 1.98 (1.66–2.36), *p* < 0.0001, *I*^2^ = 0%, *P_h_* = 0.49] mutations showed significantly shorter OS and RFS [HR = 3.11 (2.28–4.260, *p* < 0.0001, *I*^2^ = 0%, *P_h_* = 0.94] in CSF3R, [HR = 1.80 (1.14–2.84), *p* = 0.01, *I*^2^ = 7.7%, *P_h_* = 0.37] in TET2, and [HR = 2.31 (1.67–3.19), *p* < 0.0001, *I*^2^ = 0%, *P_h_* = 0.67] in TP53 AML patients with these mutations compared to those with the wild-type gene (Figure 3C–H).

### 3.6. Genes with Significant Impact on OS or RFS After Sensitivity Analysis

**Figure 4:**
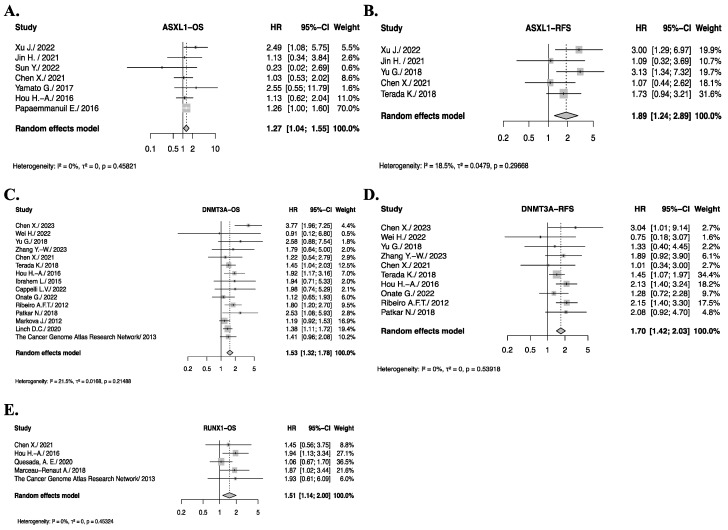
Genes with significant OS or RFS impact on de novo AML patients after sensitivity analysis. Forest plot illustrating Hazard Ratios (HR) and their corresponding confidence intervals (CI) for overall survival (OS) in de novo AML patients. (**A**), Pooled HRs and 95% CI for ASXL1 OS. (**B**), Pooled HRs and 95% CI for ASXL1 RFS. (**C**), Pooled HRs and 95% CI for DNMT3A OS. (**D**), Pooled HRs and 95% CI for DNMT3A RFS. (**E**), Pooled HRs and 95% CI for RUNX1 OS.

The pooled HRs indicated that ASXL1, DNMT3A and RUNX1 mutations were significantly associated with worse OS and RFS in de novo AML patients (Supplemental Figure S2A–F). Given the moderate heterogeneity observed across studies, we conducted a sensitivity analysis to assess the robustness of these findings. This revealed that the study by Yu G. et al. [91] contributed significantly to heterogeneity in the ASXL1 OS results. After excluding this study, the pooled HR for OS was 1.27 (1.04–1.55), *p* < 0.0098 with no residual heterogeneity (*I*^2^ = 0%, *P_h_* = 0.46) (Figure 4A). Similarly, omitting the study by Hou H.-A et al. [69] significantly reduced the heterogeneity in the pooled RFS for AML patients with ASXL1 mutations (Figure 4B). After removing this study, the pooled HR for RFS was 1.89 (1.24–2.89), *p* < 0.0030, *I*^2^ = 18.5%, *P_h_* = 0.30.

For DNMT3A mutations, the sensitivity analysis revealed that the study by Zare-Abdollahi et al. [87] was the primary source of heterogeneity in the pooled HRs for both OS and RFS. Excluding this study resulted in pooled HRs of 1.53 (1.32–1.78), *p* < 0.0001, *I*^2^ = 31%, *P_h_* = 0.12 for OS and 1.70 (1.42–2.030, *p* < 0.0001, *I*^2^ = 0%, *P_h_* = 0.54 for RFS (Figure 4C,D).

Lastly, we observed significantly poorer OS in de novo AML patients with RUNX1 somatic mutations, with a HR of 1.30 (1.03–1.63), *p* < 0.02, *I*^2^ = 32%, *P_h_* = 0.19 (Supplemental Figure S2E). The sensitivity analysis revealed that excluding the study by Papaemmanuil and colleagues [20], resulted in a pooled HR of 1.51 (1.14–2.00), *p* = 0.0044, *I*^2^ = 0%, *P_h_* = 0.45 (Figure 4E). Similarly, RUNX1 mutations showed a significantly worse impact on RFS [HR = 2.2 (1.07–4.61), *p* = 0.03], but due to the small number of studies included (*n* = 2), we were unable to conduct a sensitivity analysis to assess the source of the moderate heterogeneity (*I*^2^ = 36%). However, the heterogeneity was not statistically significant (*P_h_* = 0.2) (Supplemental Figure S2F).

### 3.7. Genes with Significant Impact on OS or RFS After Sub-Group Analysis

AML patients harboring FLT3-ITD mutations were found to have significantly shorter OS [HR = 1.70 (1.45–1.99), *p* < 0.0001, *I*^2^ = 48%, *P_h_* = 0.003] and RFS [HR = 1.62 (1.36–1.92), *p* < 0.0001, *I*^2^ = 40%, *P_h_* = 0.03] (Supplemental Figure S3A,B). Due to moderate heterogeneity observed among the selected studies, we conducted a sensitivity test and found that omitting any single study did not significantly influence the heterogeneity of OS or RFS results. Hence, subgroup analyses were proposed in Table 2 and Table 3. We assessed the pooled HRs for OS and RFS among de novo AML patients with FLT3-ITD mutations in different subgroups based on age, sample origin, and the data type used to calculate the HR. FLT3-ITD mutations were associated with poorer OS and RFS outcomes, with low heterogeneity in studies from European populations (*I*^2^ = 25% for OS, and 4% for RFS) and studies that included mixed age groups (*I*^2^ = 0% for OS, and 0% for RFS). However, no significant differences were detected between the tested subgroups (Table 2 and Table 3).

A total of 15 and 11 studies that met the inclusion criteria provided data on the effects of cKIT mutations on OS and RFS in de novo AML patients, respectively. The meta-analysis revealed that cKIT mutations were associated with a negative impact on OS [HR = 1.65 (1.13–2.41)] and RFS [HR = 1.42 (0.98–2.07)] in AML patients, but with high heterogeneity (Supplemental Figure S3C,D). Sensitivity analysis did not identify any single study that significantly influenced heterogeneity. Therefore, we performed a subgroup analysis. No significant subgroup differences were found when we assessed the pooled HRs for OS and RFS. However, the pooled HRs for both OS and RFS associated with cKIT mutations indicated worse survival outcomes with low heterogeneity among studies conducted in the adult-only age group (*I*^2^ = 0%) (Table 2 and Table 3).

The pooled OS HRs for WT1 mutations indicated a significantly shorter OS, but with substantial heterogeneity [HR = 1.65 (1.14–2.38), *p* = 0.008, *I*^2^ = 72%, *P_h_* < 0.0001] (Supplemental Figure S3E). Excluding the study by Zidan and colleagues [59], heterogeneity decreased from 72% to 41% (*I*^2^) but remained statistically significant (*P_h_* = 0.07). Based on this, we conducted a subgroup analysis and identified a significant decrease in OS with low heterogeneity in studies from the European population [HR = 1.45 (1.10–1.91), *I*^2^ = 16.8%] and in studies conducted on pediatric cohorts [HR = 1.73 (1.27–2.36), *I*^2^ = 0%]. Furthermore, we observed significant differences between studies that used multivariate analysis and those that used other methods to calculate HRs (p* = 0.01) (Table 2).

In contrast, AML patients harboring NPM1 mutations were found to have significantly longer OS [HR = 0.67 (0.51–0.88), *p* = 0.004, *I*^2^ = 69%, *P_h_* < 0.0001] (Supplemental Figure S3F). Subgroup analyses were performed, and we found that NPM1 mutations had a favorable impact on OS with a moderate heterogeneity across European [0.59 (0.38–0.93)], adults [0.76 (0.60–0.96)], and multivariate [0.61 (0.42–0.87)] subgroups. However, no significant differences were detected between the tested subgroups (Table 2).

### 3.8. Genes with Non-Significant Impact on OS or RFS of De Novo AML Patients

The pooled HRs for NRAS [HR = 0.78 (0.5–1.22), *p* = 0.3] and IDH2 [HR = 1.04 (0.6–1.8), *p* = 0.88] showed a non-significant impact of these mutations on OS (Supplemental Figure S4A,B). The subgroup analysis also revealed a non-significant impact across all tested subgroups (Supplemental Table S2). Similarly, the pooled HRs for FLT3-TKD, GATA2, IDH1, EZH2, CEBPA monoallelic (CEBPAsm), SRSF2, KRAS, SETD2, PTPN11, KMT2D, U2AF1, NOTCH1, PHF6, MLL-PTD, and RELN showed no significant impact on the OS of de novo AML patients harboring these mutations compared to patients with the respective wild-type gene (Supplemental Figure S4C–Q). It is worth noting that the number of studies included for these genes was five or fewer, so we did not conduct a subgroup analysis.

The pooled HRs for RFS of NRAS [HR = 1.22 (0.8–1.9), *p* = 0.4], WT1 [HR = 1.77 (0.9–3.3), *p* = 0.08], and NPM1 [HR = 0.65 (0.4–1.05), *p* = 0.07] showed a non-significant impact of these mutations on RFS (Supplemental Figure S5A–C). The subgroup analysis of NRAS and NPM1 revealed no significant impact across all tested subgroups (Supplemental Table S3). In contrast, WT1 mutations showed a significantly worse impact on RFS in European [*n* = 5, HR = 2.1 (1.29–3.4), *I*^2^ = 43%], pediatric [*n* = 3, HR = 2.04 (1.14–3.67), *I*^2^ = 36%], and mixed-age group cohorts [*n* = 4, HR = 2.05 (1.42–2.97), *I*^2^ = 30%], as well as in studies with data from univariate analysis or Kaplan–Meier curves [*n* = 5, HR = 3.04 (2.04–4.53), *I*^2^ = 0%] (Supplemental Table S3).

The pooled HRs for FLT3-TKD, GATA2, IDH2, EZH2, RELN, IDH1, PTPN11, KMT2D, KMT2A, and KRAS showed no significant impact on the RFS of de novo AML patients harboring these mutations compared to patients with the respective wild-type gene (Supplemental Figure S5D–M). Similarly to OS, the number of studies included for these genes was five or fewer, so we did not conduct a subgroup analysis.

### 3.9. Publication Bias

Due to the limited number of studies included for many genes, only the data on the OS and RFS of DNMT3A, FLT3-ITD, cKIT, WT1, NRAS, and the OS data of NPM1 and CEBPAdm were used to assess publication bias. No evident publication bias was observed based on the funnel plot (Supplemental Figures S6 and S7) and the *p*-values from Egger’s and Begg’s tests (Supplemental Table S4).

## 4. Discussion

Progress in sequencing technology has enabled a deeper exploration of the genetic landscape of AML. The identification of somatic gene mutations and mutational profiling has been a significant breakthrough in understanding the biological mechanisms of AML and guiding clinical decisions through risk stratification [2,96]. The patient population in this study includes individuals from diverse ethnic backgrounds, including both adults and children. Additionally, the proportion of male patients was slightly higher than females, which reflects the known gender difference in AML susceptibility. Overall, this cohort is heterogeneous but representative of the AML population.

Our pooled analysis showed that NPM1, followed by DNMT3A and then FLT3-ITD, are the most frequent somatic mutations in de novo AML patients. This finding is not only important for confirming the most frequent somatic mutations, but also for considerations of risk stratification and targeted therapy. It is also worth mentioning that many studies are already focused on those frequent mutations in their cohort.

There are still uncertainties regarding the impact of some gene mutations on the prognosis of AML. According to the 2022 ELN risk classification [2], patients with adverse-risk AML are advised to receive more aggressive treatment to improve their survival chances. This underscores the importance of personalized medicine in improving outcomes for individuals with AML.

The classification of AML increasingly relies on genomic analysis and the identification of recurrent somatic mutations. Risk stratification based on the genetic profile at diagnosis categorizes some somatic mutations as favorable, intermediate, or unfavorable groups. However, the majority of referrals fall into the intermediate risk category, where prognostic significance remains uncertain. Our study expands upon previous research by systematically evaluating the impact of these mutations on OS and RFS in de novo AML patients. Our pooled analysis showed a significant improvement in both OS and RFS in patients with biallelic CEBPA mutations compared to the wild type, confirming and consolidating previous findings in the literature [2]. However, a recent study by Tien-FM and colleagues [97] highlighted differences in the outcomes of AML patients harboring CEBPA mutations, revealing that the co-occurrence of WT1 or DNMT3A mutations, or dysregulated immune and metabolic pathways, was associated with poorer survival. These findings suggest the need for more refined, stratified analyses to improve risk classification and guide treatment decisions.

In contrast to CEBPA mutations, FLT3-ITD mutations exert a markedly adverse effect on prognosis in AML primarily due to their association with an increased risk of relapse, which translates into inferior OS outcomes [98]. Despite the negative impact of FLT3-ITD, it is classified as intermediate risk in the 2022 ELN guidelines, likely due to the availability of different FLT3 inhibitors. However, after conducting sensitivity and subgroup analyses, our findings confirm that FLT3-ITD mutations have a significant adverse effect on OS and RFS. Although our systematic review primarily includes recent studies, conducted after the introduction of FLT3 inhibitors, it still demonstrates significantly inferior outcomes, highlighting a discrepancy between our results and the current ELN risk stratification of FLT3-ITD. Nevertheless, it is important to acknowledge that our analysis lacks detailed information on the treatment regimens received by these patients. Furthermore, this remains a complex and evolving area, influenced by both the availability of treatment options and the specific context in which FLT3-ITD occurs.

Somatic mutations in the NPM1 gene are among the most common gene mutations in AML, occurring in approximately 25–30% of cases [99]. Consistent with this, our review found that NPM1 mutations were the most frequently detected somatic mutations in our cohort, with a similar frequency of 27%. Our analysis found a significant association between NPM1 mutations and improved OS. However, we observed substantial heterogeneity among the selected studies. Additionally, subgroup analysis did not identify any statistically significant differences between the tested subgroups.

NPM1 mutations often co-occur with other AML-associated mutations, particularly with FLT3-ITD, DNMT3A, IDH1/2, and NRAS. According to the current ELN recommendations for the diagnosis and treatment of AML, patients with cytogenetically normal AML who carry an NPM1 mutation but without FLT3-ITD mutations are categorized as favorable risk. Detailed subgroup analysis of co-occurring mutations is beyond the scope of this meta-analysis but would be beneficial to further stratify patients with NPM1 mutations based on these additional genetic factors.

AML patients with DNMT3A mutations are more commonly found in older patients and are associated with higher white blood cell count. These patients are also more likely to have monocytic leukemia. Several studies have reported a negative prognostic impact of DNMT3A mutations based on other factors such as cytogenetics and other associated mutations like FLT3 and NPM1 [100]. In our review after conducting the sensitivity analysis, we found a significantly poorer outcome for both OS and RFS, confirming the independent negative impact of DNMT3A mutation.

cKIT mutations occur in 20–25% of t(8; 21) cases and in approximately 30% of inv(16) cases [36]. While several studies have reported an adverse impact of cKIT mutations on prognosis, others have shown no significant impact or have suggested that the negative impact is limited to t(8; 21) cases only [101]. Our findings indicate that cKIT mutations have an adverse effect on OS and RFS, particularly in adults, as demonstrated in our subgroup analysis. The National Comprehensive Cancer Network (NCCN) guidelines [102] and ELN both classify cKIT mutation as intermediate risk. However, our findings suggest that further subgroup analyses might be necessary for better risk stratification among pediatric and adult patients. Moreover, the subgroup analysis detected a regional difference in the prognostic impact of c-KIT mutations, where the association between c-KIT mutations and poorer outcomes was notable in non-European cohorts for both OS and RFS. The subgroup difference did not reach the statistically significant threshold, but the moderate heterogeneity observed within subgroups suggests that regional or population-specific factors may result in these differences. These findings highlight the need for further large-scale and region-specific studies to clarify the prognostic relevance of c-KIT mutations across different populations.

The RAS gene family consists of three homologues: HRAS, KRAS, and NRAS. In our review, we found that NRAS mutations were found to be more prevalent in pediatric patients. Despite being identified nearly 30 years ago, the prognostic implications of RAS mutations in AML remain a topic of debate. Several studies suggest that RAS mutations have no significant impact on prognosis in AML patients with normal cytogenetics. However, other studies have reported contradicting results, linking RAS mutations to poorer outcomes in AML [103]. Interestingly, in pediatric AML, activating NRAS mutations are commonly observed, often in conjunction with NPM1 mutations, particularly within the favorable risk population. Neither in pooled analysis nor in sensitivity analysis could we find a significant impact of NRAS or KRAS on OS or RFS in de novo AML cases; thus, the prognostic meaning of these mutations is still questionable and requires further studies.

There is a need for more clarity about the prognostic consequences of somatic mutations like those affecting genes such as GATA2, FLT3-TKD, IDH1, IDH2, EZH2, CEBPAsm, and others. These mutations are relatively infrequent, and their significance to risk-stratified therapeutic regimens is still unknown [2]. On the other hand, our study reinforces previous findings on the poor prognosis associated with mutations in TP53, CSF3R, RUNX1, ASXL1, WT1, and TET2 in AML patients.

Available data is variable and can be limited for certain genes. In some studies, the only available data sources for hazard ratios were univariate analyses or Kaplan–Meier plots. The utilization of derived data in our research may introduce inherent biases and inaccuracies in our conclusions. Furthermore, co-occurring mutations can have a significant impact on AML prognosis, e.g., NPM1 with cKIT, or triple mutation AML with NPM1, DNMT3A, and FLT3-ITD mutations [104]. In our study, such co-occurrence could not be extracted from the included studies, and, thus, subgroup analysis could not be performed. Moreover, the prognostic significance of somatic mutations may differ between pediatric and adult patients. We observed differences in the frequency distribution of somatic gene mutations across age groups. Although we performed subgroup analyses based on age for specific genes, the inclusion of pediatric patients in the pooled analysis may have influenced the overall results. As a result, these limitations should be considered when interpreting our findings, and subsequent investigations should aim to enhance data accessibility and methodological approaches to provide more robust conclusions regarding the prognostic significance of somatic mutations in AML.

## 5. Conclusions

In conclusion, our study investigated the frequency of somatic mutations and provided strong evidence supporting their prognostic significance in a clinical context. These findings contribute to a better understanding of AML biology and can help in improving risk stratification strategies for improved patient management.

## Figures and Tables

**Table 1:** Characteristics of the studies included in the systematic review and meta-analysis.

Study ID	Country of Origin	Sample Size	M	F	U	Age in Years (Range)	Follow up in Months	Type of Molecular Test	Tested Somatic Mutations	Accompanied Somatic Mutations	NOS Score
Abbas S./2010	The Netherlands	895	429	466	0	NA (15–77)	33.2	Direct sequencing	IDH1 ^U^, IDH2 ^U^	FLT3-ITD, FLT3-TKD, NPM1, NRAS, KRAS	6
Al-Arbeed I.F./2021	Syria	44	23	21	0	35.3 ± 12.4	14	RFLP-PCR	FLT3-ITD ^K^	NA	5
Aly R.M./2011	Egypt	39	21	18	0	7.4 (5.6–13)	32 ± 2.24	PCR-SSCP	NRAS ^K^	NA	7
Aref S./2014	Egypt	153	75	78	0	NA (17–65)	48	Direct sequencing	TERT ^M^	NA	6
Aref S./2020	Egypt	50	26	24	0	NA (24–59)	24	Direct sequencing	NOTCH1 ^M^	NA	8
Awad M.M./2013	Egypt	55	25	30	0	45.65 ± 16	NA	PCR-SSCP	CEBPAsm ^K^, CEBPAdm ^K^	NA	6
Bachas C./2014	Germany and The Netherlands	198	122	76	0	10.2 (0.4–19.5)	40.6 (3.9–159.3)	HRM analysis	FLT3-ITD ^U^, FLT3-TKD ^U^, KRAS ^U^, WT1 ^U^, cKIT ^U^, NRAS ^U^, NPM1 ^U^	NA	7
Boissel N./2010	France	205	90	115	0	48 (17–70)	NA	Direct sequencing	IDH2 ^M^	FLT3-ITD, CEBPAsm, IDH1, WT1, NPM1	7
Cairoli R./2013	Italy	58	40	18	0	42 (15–60)	50	Direct sequencing	cKIT ^M^	NA	8
Canaani J./2018	Israel	293	172	121	0	NA (18–73.8)	NA	NA	FLT3-ITD ^M^	NA	8
Cappelli L.V./2022	Germany	150	73	77	0	57 (19–82)	39.6 (2.4–104.4)	WGS or NGS panel (63 genes)	FLT3-ITD ^U^, DNMT3A ^U^	NA	7
Chen X./2012	China	127	67	60	0	NA (0.3–15)	NA	Direct sequencing	WT1 ^U^	FLT3-ITD, NPM1, CEBPA, cKIT	5
Chen X./2018	China	50	31	19	0	7	32 (1–90)	Direct sequencing	cKIT ^M^	WT1, CEBPA	8
Chen X./2021	China	204	103	101	0	54.4 (20–86)	NA	NGS panel (22 genes)	NPM1 ^M^, CEBPAdm ^M^, TET2 ^M^, ASXL1 ^M^, cKIT ^M^, IDH1 ^M^, IDH2 ^M^, DNMT3A ^M^, RUNX1 ^M^, TP53 ^M^, PHF6 ^M^, NRAS ^M^	FLT3-ITD, DNMT3A	8
Chen X./2023	China	171	90	81	0	53 (19–86)	47	NGS panel (34 genes)	DNMT3A ^M^	NPM1, BCOR, FLT3-ITD, CEBPAsm, NRAS, TET2	9
Chou W.-C./2011	Taiwan	486	274	212	0	51.5 (15–90)	NA	Direct sequencing	TET2 ^M^, CEBPAdm ^M^, NPM1 ^M^	cKIT, KRAS, NRAS, FLT3-ITD, MLL-PTD, ASXL1, FLT3-TKD, WT1, RUNX1	8
Christen F./2019	Multicenter	331	188	143	0	41.7 (15–84)	80.88 (3–253.2)	NGS panel (66 genes)	RAD21 ^M^, JAK2 ^M^	ASXL1, ASXL2, CBL, DHX15, EZH2, FLT3-ITD, cKIT, KRAS, NRAS, TET2	7
Duan W./2021	China	215	124	91	0	39 (15–70)	26 (7–12)	TaqMan based RT-PCR	cKIT ^M^	FLT3-ITD, CEBPAsm, NPM1, EV11, MLL-PTD	8
Elghannam D.M./2009	Egypt	150	110	40	0	55 (19–74)	NA	PCR-SSCP	NRAS ^M^	NA	7
Guan W./2021	China	207	115	92	0	45.4 (14–76)	NA	NGS panel	FLT3-ITD ^K^, FLT3-TKD ^K^	NPM1, DNMT3A, RUNX1, KIT, PTPN11. TET2, CEBPAdm, ASXL1, TP53	9
Han H./2023	China	878	466	412	0	44 (8–78)	45.2	2 NGS panel (51 or 172 genes panels)	NOTCH ^M^	CBL, CSMD1, FLT3-TKD, JAK, PTPN11, STAG2, ZRSR2, TET2, TP53, WT1, CSF3R, FLT3-ITD, IDH2, NPM1, SETD2, CEBPAdm, EZH2, IDH1, MPL, RUNX1, CEBPAsm, DNMT3A, GATA2, cKIT, RAS	8
Hollink I. H. I./2009	Multicenter	232	133	99	0	9.6	52	Direct sequencing	FLT3-ITD ^M^, WT1 ^M^	cKIT, NPM1, MLL-PTD	7
Hou H.-A./2016	Taiwan	500	285	215	0	51 (15–90)	55 (1–160)	Direct sequencing	CEBPAdm ^M^, RUNX1 ^M^, WT1 ^M^, ASXL1 ^M^, IDH2 ^M^, DNMT3A ^M^, TP53 ^M^	cKIT, JAK2, NPM1, MLL-PTD, SF3B1, U2AF1, SRSF2, FLT-ITD, FLT3-TKD, NRAS, KRAS, PTPN11	9
Ibrahem L./2015	Egypt	120	66	54	0	47 (33–60)	30	Cycle sequencing	DNMT3A ^M^	NA	8
Ishikawa Y./2020	Japan	199	125	74	0	41 (16–64)	52.2 (11.9–81.8)	NGS panel	cKIT ^M^, TET2 ^M^, NRAS ^M^	FLT3-ITD, FLT3-TKD, KRAS, JAK2, PTPN11, ASXL1, BCORL1, EZH2, KDM6A, SMC, SMC1A, RAD21, RUNX1, WT1, CSRF3R, ASXL2, DNMT3A, ETV6	7
Jin H./2021	China	62	33	29	0	49.5 (19–83)	21.5	NGS Targeted deep sequencing	cKIT ^U^, NRAS ^M^, ASXL1 ^U^, FLT3-ITD ^U^, TET2 ^U^	DMNT3A	5
Kaburagi T./2023	Japan	369	194	175	0	7 (0–17)	NA	NGS panel (343 genes) or Direct sequencing	UBTF-ITD ^K^	FLT3-ITD, WT1	5
Koczkodaj D./2022	Poland	90	42	48	0	62.63 (18–85)	NA	Direct sequencing	FLT3-ITD ^M^, WT1 ^M^, NPM1 ^M^	NA	7
Kurosawa S./2020	Japan	235	141	94	0	51 (18–65)	NA	NGS (Ion torrent) + Direct sequencing	FLT3-ITD ^M^, NPM1 ^U^, CEBPAdm ^M^	NA	8
Linch D.C./2020	UK	876	368	508	0	NA (16–59)	108 (4–260)	Capillary electrophoresis	FLT3-ITD ^U^, DNMT3A ^U^	NA	7
Marceau-Renaut A./2018	France	385	210	175	0	8.6 (0–18)	59	NGS panel (36 genes)	NPM1 ^M^, FLT3-ITD ^K^, CEBPAdm ^M^, WT1 ^M^, RUNX1 ^M^, PHF6 ^M^	FLT3-TKD, cKIT, NRAS, KRAS, CBL, TET2, PTPN11, ASXL1, SMC1A, SF3B1, JAK2, EZH2, SMC3, ZRSR2, MPL, BCOR, RAD21, U2AF1, SETBP1, BCORL1, STAG2, DNMT3A, GATA2, IDH1, IDH2, ETV6, TP53, NPM1, WT1, GATA1, GATA2	7
Markova J./2012	Czech Republic	226	107	119	0	54.9 (18.2–81.7)	1.6 (0–202)	Direct sequencing	DNMT3A ^K^	NA	7
Mason EF./2019	US	239	110	129	0	64.8 (14–89)	14.2 (0.1–88.4)	NGS panel (95 genes)	FLT3-ITD ^M^	NPM1, SRSF2, IDH2, TET2, IDH1, DNMT3A, RAS, WT1	8
Matsuo H./2020	Japan	160	72	88	0	3.9 (0.0–18.2)	NA	NGS panel (338 genes)	FLT3-ITD ^M^, KRAS ^M^, NRAS ^M^, PTPN11 ^M^, SETD2 ^M^, STAG2 ^M^, CCND3 ^M^, U2AF1 ^M^	NA	8
Mechaal A./2019	Tunisia	211	110	101	0	35 (2–80)	NA	Direct sequencing	EZH2 ^K^	IDH2, NPM1, FLT3-ITD, DNMT3A	5
Metafuni E./2022	Italy	96	57	39	0	56 (17–73)	NA	NGS panel (26 gene)	TP53 ^M^, NRAS ^M^, WT1 ^M^, FLT3-ITD ^M^	ASXL1, cKIT, DNMT3A, EZH2, TET2, SRSF2, RUNX1, KRAS, IDH1, IDH2, U2AF1	6
Moualla Y./2022	Syria	100	51	49	0	NA	NA	Direct sequencing	FLT3-ITD ^M^	FLT3-TKD	6
Nakamura H./2004	Japan	24	12	12	0	54 (34–78)	9 (6–81)	Direct sequencing	NRAS ^K^, TP53 ^K^, FLT3-ITD ^K^	NA	9
Onate G./2022	Spain	164	58	106	0	NA (18–72)	30	Direct sequencing	DNMT3A ^K^	NA	8
Papaemmanuil E./2016	UK	1540	823	717	0	54 (18–84)	70.8 (1–179)	NGS panel (111 genes)	FLT3-ITD ^M^, GATA2 ^M^, TP53 ^M^, BRAF ^M^, SRSF2 ^M^, NPM1 ^M^, CEBPAdm ^M^, ASXL1 ^M^, ZRSR2 ^M^, RUNX1 ^M^, IDH2 ^M^	DNMT3A, FLT3-TKD, STAG2, RAD21	8
Park SH./2013	South Korea	157	91	66	0	50.65 ± 17.2	NA	Direct sequencing	FLT3-ITD ^M^, CEBPAsm ^M^, CEBPAdm ^M^, NPM1 ^M^	DNMT3A, IDH1, IDH2	8
Paschka et al. 2006	US	110	59	51	0	NA	64	Direct sequencing and DHLPC	cKIT ^M^	NA	9
Patkar N./2018	India	83	46	37	0	36.7 (18–62)	23.5	NGS Targeted deep sequencing	FLT3-ITD ^U^, DNMT3A ^U^	NPM1	7
Pollard J.A./2010	US	203	106	97	0	NA (0.6–19.6)	66.8 (1.9–104.5)	Direct sequencing	cKIT ^K^	FLT3-ITD, WT1	8
Pratcorona M./2013	Spain	303	173	130	0	47 (17–60)	NA	Direct sequencing	FLT3-ITD ^M^, NPM1 ^M^	NA	8
Quesada, A. E./2020	US	140	79	61	0	NA (20–87)	21.4	Direct sequencing	RUNX1 ^U^, NPM1 ^U^	FLT3-ITD, NRAS, IDH2, SRSF2, EZH2, DNMT3A, CEBPA, TET2, ASXL1	9
Ribeiro A.F.T./2012	The Netherlands	415	210	205	0	41 (15–60)	115.7 (7.2–224.1)	HPLC	DNMT3A ^M^, FLT3-ITD ^M^, NPM1 ^M^, CEBPAdm ^M^, NRAS ^M^, IDH1 ^M^, IDH2 ^M^, EV11 ^M^, WT1 ^M^, cKIT ^M^	NA	8
Riera L./2013	Italy	23	11	12	0	42.7 (19–64)	88	Direct sequencing	cKIT ^K^	NA	6
Sakaguchi M./2018	Japan	147	66	77	4	56 (18–90)	11.5	Fragment analysis	NPM1 ^U^	CEBPAsm, CEBPAdm, FLT3-ITD	8
Sakaguchi M./2019	Japan	674	395	276	3	57 (15–94)	NA	RFLP-PCR	FLT3-ITD ^K^, FLT3-TKD ^K^	NPM1, CEBPAsm, CEBPAdm	7
Sengsayadeth S.M./2012	US	75	37	38	0	49 (20–68)	NA	NA	FLT3-ITD ^K^	NA	8
Shimada A./2008	Japan	158	89	69	0	6 (0–15)	NA	Direct sequencing	FLT3-ITD ^M^, MLL-PTD ^M^	NA	7
Shouval R./2020	France	405	200	205	0	52.5 (42.9–60)	66 (43.2–93.6)	NA	FLT3-ITD ^K^, NPM1 ^K^	NA	8
Su L./2018	China	81	45	36	0	44 (9–67)	8 (2–66)	NGS panel (112 genes)	CSF3R ^K^, WT1 ^K^, GATA2 ^K^	NRAS, TET2, CEBPAdm	6
Su L/2019	China	101	53	48	0	43 (9–79)	18.5 (3–78)	Direct sequencing	CSF3R ^K^	NA	8
Sun Y./2022	China	74	35	39	0	43 (6–68)	NA	NGS panel	ETV6 ^M^, TET2 ^M^, ASXL1 ^M^	PTPN11, DNMT3A, NPM1, CEBPA, FLT3-ITD, EZH2, NRAS, cKIT	8
Suzuki T./2005	Japan	190	NA	NA	190	50 (15–85)	NA	Direct sequencing	NPM1 ^K^	FLT3-ITD, TP53, NRAS	7
Tarlock K./2019	US	205	103	102	0	11.5 (0.33–22.76)	64. 9 (0–96.9)	Targeted exome capture sequencing	cKIT ^U^	FLT3-ITD	7
Terada K./2018	Japan	412	235	174	3	55.1 (15–91)	NA	NGS Ion PGM™ and Direct sequencing	TP53 ^M^, FLT3-ITD ^M^, TET2 ^M^, DNMT3A ^M^, NRAS ^M^, cKIT ^M^, CEBPAdm ^M^, MLL-PTD ^M^, ASXL1 ^M^	NOTCH1, NCOR2, IDH2, WT1, CEBPAsm, IDH1, PTPN11, GATA2, BCOR, NPM1, BCORL1, FLT3-TKD	8
The Cancer Genome Atlas Research Network/2013	US	200	108	92	0	55.0 ± 16.1	NA	WGS or WES	TP53 ^M^, DNMT3A ^M^, FLT3-ITD ^M^, RUNX1 ^M^	NPM1, TET2, CEBPAdm, WT1, PTPN11, KIT	8
Tien F.-M./2018	Taiwan	693	395	298	0	55 (15–94)	78.6 (0.1–236)	Ion torrent NGS and Direct sequencing	GATA2 ^M^, CEBPAdm ^M^	IDH1, IDH2, NPM1, TET2, CEBPAsm, DNMT3A, KRAS, WT1, PTPN11, NRAS, ETV6, RUNX1, MLL-PTD, TP53, cKIT, ASXL1, FLT3-ITD, FLT3-TKD	7
Toogeh G./2016	Iran	88	55	33	0	42 ± 12	24	Direct sequencing	WT1 ^K^	NA	8
Virijevic M./2016	Serbia	110	62	48	0	53.5 (19–78)	NA	Direct sequencing	FLT3-ITD ^M^	NA	8
Wakita S./2016	Japan	271	157	114	0	54. (17–86)	NA	NGS (Ion torrent)	NPM1 ^M^, FLT3-ITD ^M^, CEBPAdm ^M^, NRAS ^M^	CEBPAsm, IDH1, IDH2, FLT3-TKD, KMT2A, KRAS, TET2, DNMT3A, ASXL1, KMT2A, RUNX1, cKIT, TP53, PTPN11, GATA2, WT1, STAG2, SMC1A, SMC3, DAXX, BCOR, BCORL1, NF1, DDX41, PHF6	9
Wakita S./2022	Japan	1028	580	448	0	54.3 (16–70)	NA	Direct sequencing	CEBPAdm ^K^, CEBPAsm ^K^	FLT3-ITD, NPM1	8
Wang J./2020	China	124	73	51	0	37.5 (16–69)	33.5 (4–69)	NGS panel (87 genes)	NRAS ^K^, WT1 ^K^, GATA2 ^K^, CSF3R ^K^	FLT3-ITD	7
Wang T./2022	China	220	114	106	0	39 (18–88)	30.5 (0.5–60.6)	NGS panel (112 genes) and Direct sequencing	WT1 ^K^	CEBPAdm, RUNX1, IDH1, JAK2, CSF3R, ZRSR2, SMC3, SRSF2, SF3B1, RAD21, BCOR, BCORL, cKIT, TP53, ASXL1, FAT1, EZH2, GATA2, IDH2, SH2B3, RELN, NRAS, SETBP1, DNMT3A, PTPN11, NOTCH11, KRAS, CEBPAsm, ETV6, TET2, NOTCH2, FLT3-ITD	7
Wei H./2022	China	171	100	71	0	38 (14–59)	39 (0.3–106)	Direct sequencing and NGS panel (69 genes)	CSF3R ^M^, WT1 ^M^, CUX1 ^M^, GATA2 ^M^, NRAS ^M^, FLT3-ITD ^M^, JAK3 ^M^, TET2 ^M^, CREBBP ^M^, cKIT ^M^, NOTCH1 ^M^, KMT2D ^M^, DNMT3A ^M^, EZH2 ^M^, EP300 ^M^, NOTCH2 ^M^, RELN ^M^, SH2B3 ^M^	NA	8
Wu L.-X./2021	China	158	55	103	0	41 (17–74)	NA	NGS panel (236 genes)	NRAS ^U^, PCLO ^U^, KMT2A ^M^, CSF3R ^M^	GATA2, WT1, TET2, FLT3-ITD, DNMT3A, BAZ2A, NPM1, AHNAK2	8
Xu J./2022	China	156	82	74	0	NA	NA	NGS panel (34 genes)	ASXL1 ^M^	TET2, CBL, TP53, SH2B3, CEBPAsm, DNMT3A, FLT3-ITD, NPM1, JAK2, CSF3R, cKIT, U2AF1, GATA2, PHF6, SRSF2, ETV6, MPL	7
Xu N./2022	China	84	46	38	0	54 (18–69)	10 (1–102)	Bidirectional sequencing on an ABI 3730 sequencer	TP53 ^M^	FLT3-ITD, NPM1, CEBPAdm	5
Yamato G./2017	Japan	369	179	190	0	8.4 (0–17.9)	36	NGS Targeted deep sequencing	ASXL1 ^K^, ASXL2 ^K^	FLT3-ITD, NRAS, WT1, KMT2A, BCOR, BCORL1, STAG2, CSF3R, SMC3, CEBPA	8
Yang J./2016	China	249	145	104	0	NA (18–93)	NA	Direct sequencing	SRSF2 ^M^, U2AF1 ^M^	cKIT, IDH1, IDH2, NPM1, DNMT3A, FLT3-ITD, CEBPA, SF3B1	8
Yu G./2018	China	64	39	25	0	27.5 (2–65)	23.5 (4–85)	NGS panel (67 genes)	cKIT ^M^, ASXL1 ^M^, MET ^U^, MLH1 ^U^, TET2 ^U^, FBXW7 ^U^, TP53 ^U^, DNMT3A ^U^, KMT2A ^U^, PAX5 ^U^	NRAS, APC, RUNX1, NPM1, KRAS, SH2B3, HRAS, SMAD4, DNMT3L	6
Yuen K.-Y./2023	China	493	255	238	0	8.7	55	NGS panel (177 genes)	KRAS ^M^, FLT3-ITD ^M^, NRAS ^M^, SETD2 ^M^	FLT3-TKD, WT1, cKIT	9
Yui S/2017	Japan	136	91	45	0	45 (15–80)	NA	Direct sequencing	cKIT ^M^	NA	8
Zare-Abdollahi D./2015	Iran	96	53	43	0	42 (18–60)	33	Direct sequencing	DNMT3A ^M^	NA	7
Zhang Y.-W./2023	China	266	114	152	0	52.5 (12–78)	26.0 (4.0–101.3)	NGS panel (112 genes)	DNMT3A ^M^, TET2 ^M^, FLT3-TKD ^M^, PTPN11 ^M^, FLT3-ITD ^M^	NPM1, IDH, RAS	7
Zhong WJ/2021	China	113	66	47	0	56 (18–89)	15 (1–54)	NGS panel (141 genes)	SRSF2 ^K^, KRAS ^K^, KMT2D ^K^, FAT1 ^K^, RELN ^K^	FLT3-ITD, NPM1, SETBP1, NOTCH11, ASXL1, NRAS, DNMT3A, CUX1, TET2, JAK2, RUNX1, ATM, TP53, cKIT, CREBBP, CARD11, SH2B3, BRAF, DIS3, DDX41, BCORL1, DNMT3B, FGFR3, ARID1A	8
Zidan M./2014	Egypt	216	109	107	0	44.16 ± 15.7	NA	PCR-SSCP	WT1 ^M^, NPM1 ^M^	NA	9

Abbreviations: NGS, Next Generation Sequencing; CEBPAdm, CEBPA biallelic; CEBPAsm, CEBPA monoallelic; HPLC, High-performance liquid chromatography; HRM, High Resolution Melting; RFLP, Restriction Fragment Length Polymorphism; DHLPC, Denaturing high-performance liquid chromatography; M, multivariate; U, univariate; K, Kaplan–Meier curve; NOS, Newcastle–Ottawa Quality Assessment Scale; NA, Not available.

**Table 2:** Subgroup analyses of OS on somatic gene mutations impact in de novo AML patients.

COMPARISON VARIABLES	FLT3-ITD				cKIT				WT1				NPM1			
	K	HR (95%CI)	*I*^2^ (%), *P_h_*	p*	K	HR (95%CI)	*I*^2^ (%), *P_h_*	p*	K	HR (95%CI)	*I*^2^ (%), *P_h_*	p*	K	HR (95%CI)	*I*^2^ (%), *P_h_*	p*
**TOTAL**	28	1.70 (1.45–1.99)	48%, 0.003	<0.0001	15	1.65 (1.13–2.41)	72%, <0.0001	0.002	13	1.65 (1.14–2.38)	72%, 0.008	0.01	16	0.67 (0.51–0.88)	69%, <0.001	0.01
**REGION**																
**EUROPEAN**	10	1.67 (1.47–1.89)	25%	0.8	4	0.93 (0.46–1.89)	60%	0.05	5	1.45 (1.10–1.91)	17%	0.44	7	0.59 (0.38–0.93)	58.5%	0.23
**NON-EUROPEAN**	18	1.73 (1.36–2.21)	56%		11	2.04 (1.46–2.87)	59%		8	1.89 (1.04–3.44)	81%		9	0.83 (0.61–1.13)	61%	
**AGE GROUP**																
**PEDIATRIC**	5	1.55 (0.92–2.60)	61%	0.6	3	1.58 (0.50–4.98)	79%	0.1	4	1.73 (1.27–2.36)	0%	0.97	2	0.33 (0.06–1.95)	78%	0.57
**ADULT**	15	1.66 (1.26–2.17)	59%		5	2.74 (1.84–4.08)	0%		5	1.69 (0.63–4.54)	87%		11	0.76 (0.60–0.96)	56%	
**MIXED**	8	1.87 (1.68–2.10)	0%		7	1.4 (0.86–2.28)	65%		4	1.84 (1.26–2.68)	24%		3	0.60 (0.28–1.30)	86%	
**DATA TYPE**																
**MULTIVARIATE**	16	1.68 (1.40–2.01)	47%	0.9	11	1.85 (1.24–2.76)	66%	0.47	8	1.25 (0.82–1.91)	75%	0.01	10	0.61 (0.42–0.87)	46%	0.12
OTHERS *	12	1.71 (1.28–2.30)	48%		4	1.36 (0.65–2.86)	64%		5	2.64 (1.79–3.88)	0%		6	0.9 (0.64–1.25)	79%	

Abbreviations: K, number of studies; p*, Test for subgroup differences (random effects model); Others *, include univariate analysis or data from Kaplan–Meier Curve.

**Table 3:** Subgroup analyses of RFS on somatic gene mutations’ impact in de novo AML patients.

COMPARISON VARIABLES	FLT3-ITD				cKIT			
	K	HR (95%CI)	*I*^2^ (%), *P_h_*	p*	K	HR (95%CI)	*I*^2^ (%), *P_h_*	p*
**TOTAL**	20	1.62 (1.36–1.92)	40%, 0.03	<0.0001	11	1.42 (0.98–2.07)	69%, <0.0001	0.06
**REGION**								
**EUROPEAN**	8	1.62 (1.32–1.98)	4%	1	2	0.85 (0.40–1.78)	29%	0.15
**NON-EUROPEAN**	12	1.61 (1.26–2.05)	53%		9	1.59 (1.06–2.38)	71%	
**AGE GROUP**								
**PEDIATRIC**	4	1.64 (0.88–3.05)	71%	0.6	3	0.77 (0.46–1.30)	63%	0
**ADULT**	10	1.54 (1.16–2.04)	39%		3	2.65 (1.69–4.16)	0%	
**MIXED**	6	1.80 (1.54–2.11)	0%		5	1.81 (1.22–2.69)	19%	
**DATA TYPE**								
**MULTIVARIATE**	13	1.73 (1.42–2.10)	35%	0.3	7	2.02 (1.41–2.90)	29%	0.03
OTHERS *	7	1.43 (1.05–1.96)	49%		4	0.94 (0.52–1.69)	68%	

p*: Test for subgroup differences (random effects model), Others *, include univariate analysis or data from Kaplan–Meier Curve.

## Data Availability

All data generated or analyzed during this study are included in this published article and its Supplementary Information files.

## Supplementary Materials

The following supporting information can be downloaded at: https://www.mdpi.com/article/10.3390/cancers17193189/s1, Figure S1: Bar plot displays the frequency distribution of somatic gene mutations among pediatric de novo AML patients. Each bar on the x-axis corresponds to a specific gene with a mutation, whereas the y-axis illustrates the frequency of these mutations across included studies; Figure S2: Genes linked to significant overall survival or relapse free survival outcomes in de novo AML patients evaluated by sensitivity analysis. Forest plot illustrating hazard ratios (HR) and their corresponding confidence intervals (CI) for overall survival (OS) or relapse free survival (RFS) in de novo AML patients. Studies enclosed in red boxes indicate the greatest source of heterogeneity; Figure S3: Genes linked to significant overall survival and relapse free survival outcomes in de novo AML patients prior to subgroup analysis evaluation. Forest plot illustrating hazard ratios (HR) and their corresponding confidence intervals (CI) for overall survival (OS) or relapse free survival (RFS) in de novo AML patients; Figure S4: Genes with Non-significant OS impact on de novo AML patients. Forest plot illustrating hazard ratios (HR) and their corresponding confidence intervals (CI) for overall survival (OS) in de novo AML patients; Figure S5: Genes with Non-significant RFS impact on de novo AML patients. Forest plot illustrating hazard ratios (HR) and their corresponding confidence intervals (CI) for relapse free survival (RFS) in de novo AML patients; Figure S6: Funnel plot for the publication bias test of the tested genes mutations in OS; Figure S7: Funnel plot for the publication bias test of the tested genes mutations in RFS; Table S1: The Prognostic impact of somatic gene mutations on over-all survival and relapse free survival of de novo AML patients; Table S2: Subgroup analyses of OS on somatic genes mutations impact in de novo AML patients; Table S3: Subgroup analyses of RFS on somatic genes mutations impact in de novo AML patients; Table S4: Egger’s and Begg’s tests for publication bias.
