# Supplementary material for: Impact of Somatic Gene Mutations on Prognosis Prediction in De Novo AML: Unraveling Insights from a Systematic Review and Meta-Analysis
Source: Cancers (Basel). 2025 Sep 30;17(19):3189. doi: 10.3390/cancers17193189 (PMC12523279; doi:10.3390/cancers17193189)
Supplement: Supplementary file 1 [file cancers-17-03189-s001.zip › cancers-3839614-supplementary.pdf]

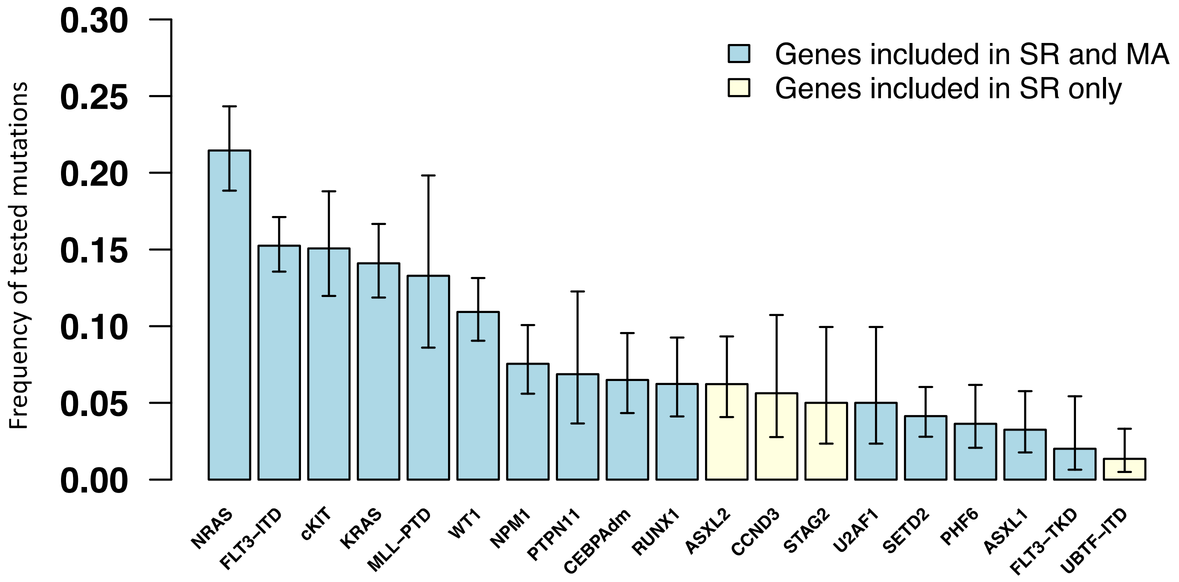

Supplementary Figure S1. Bar plot displays the frequency distribution of somatic gene mutations among pediatric de novo AML patients. Each bar on the x-axis corresponds to a specific gene with a mutation, whereas the y-axis illustrates the frequency of these mutations across included studies.

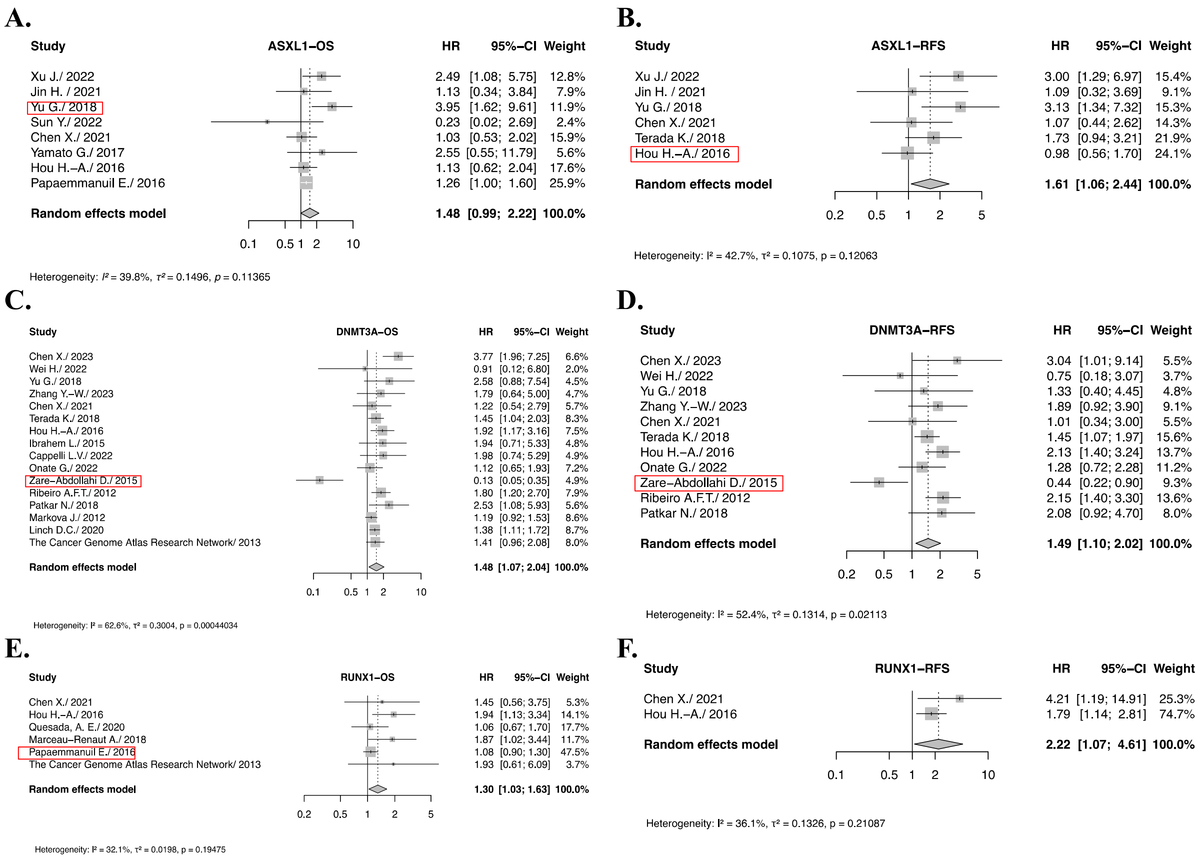

Supplementary Figure S2. Genes linked to significant overall survival or relapse free survival outcomes in de novo AML patients evaluated by sensitivity analysis. Forest plot illustrating hazard ratios (HR) and their corresponding confidence intervals (CI) for overall survival (OS) or relapse free survival (RFS) in de novo AML patients. Studies enclosed in red boxes indicate the greatest source of heterogeneity.

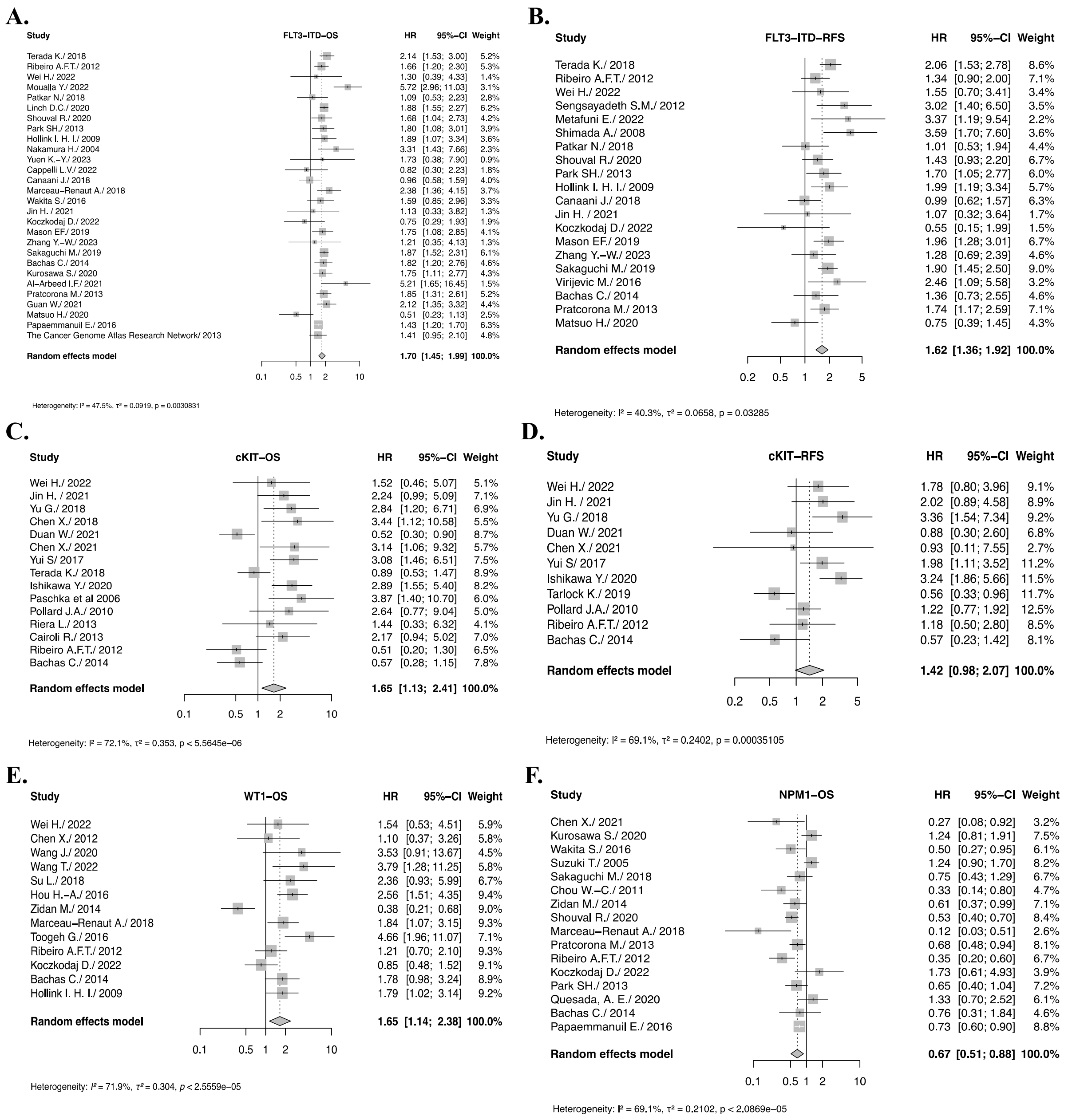

Supplementary Figure S3. Genes linked to significant overall survival and relapse free survival outcomes in de novo AML patients prior to subgroup analysis evaluation. Forest plot illustrating hazard ratios (HR) and their corresponding confidence intervals (CI) for overall survival (OS) or relapse free survival (RFS) in de novo AML patients.

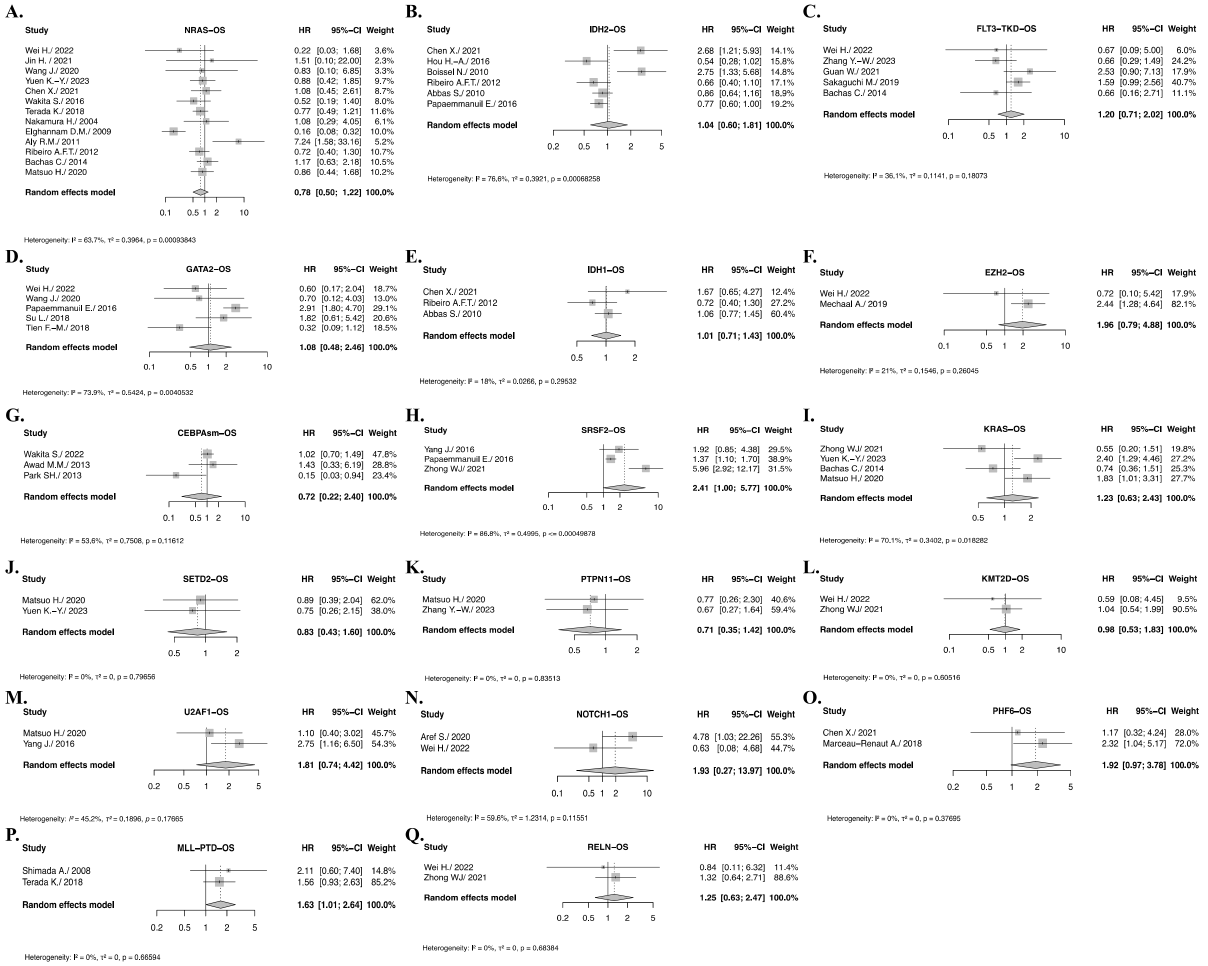

Supplementary Figure S4. Genes with Non-significant OS impact on de novo AML patients. Forest plot illustrating hazard ratios (HR) and their corresponding confidence intervals (CI) for overall survival (OS) in de novo AML patients.

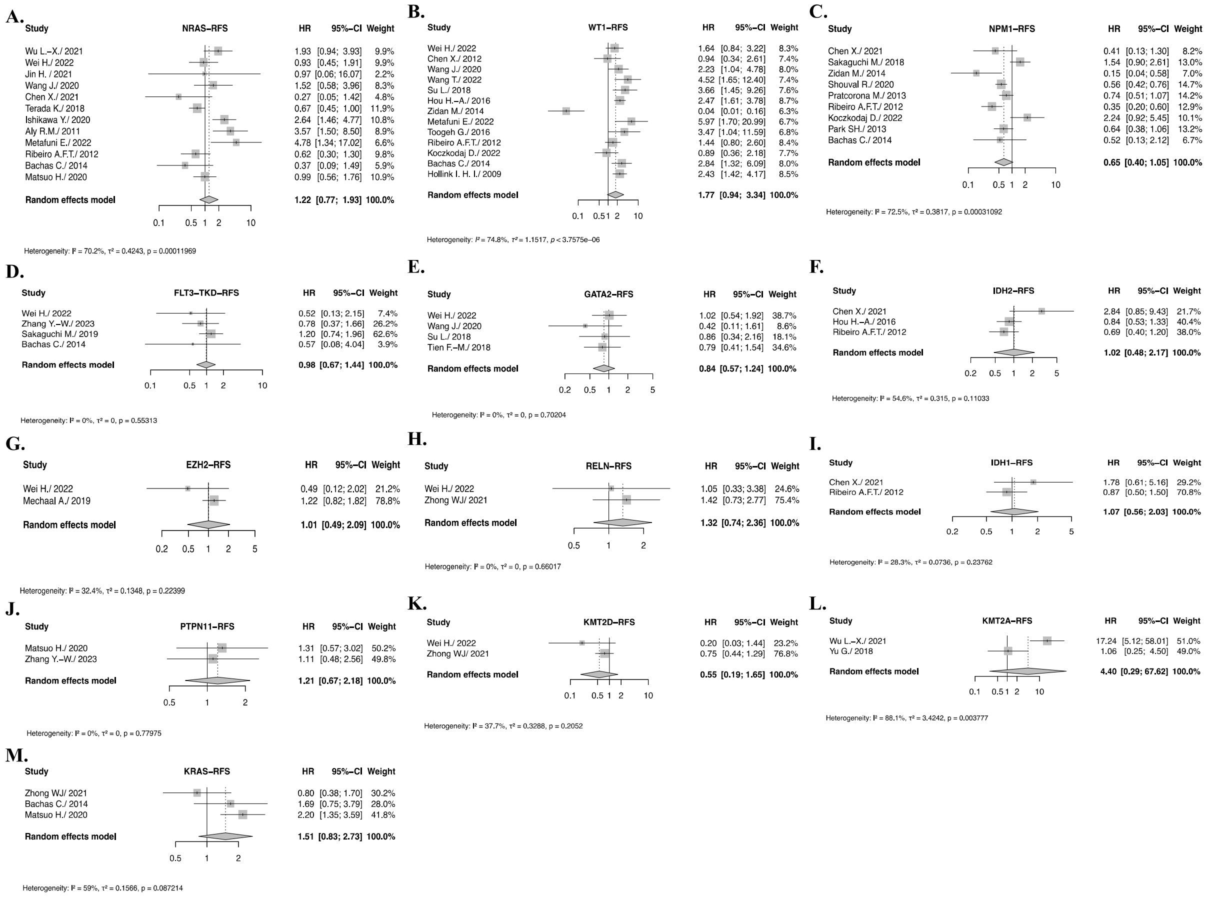

Supplementary Figure S5. Genes with Non-significant RFS impact on de novo AML patients. Forest plot illustrating hazard ratios (HR) and their corresponding confidence intervals (CI) for relapse free survival (RFS) in de novo AML patients.

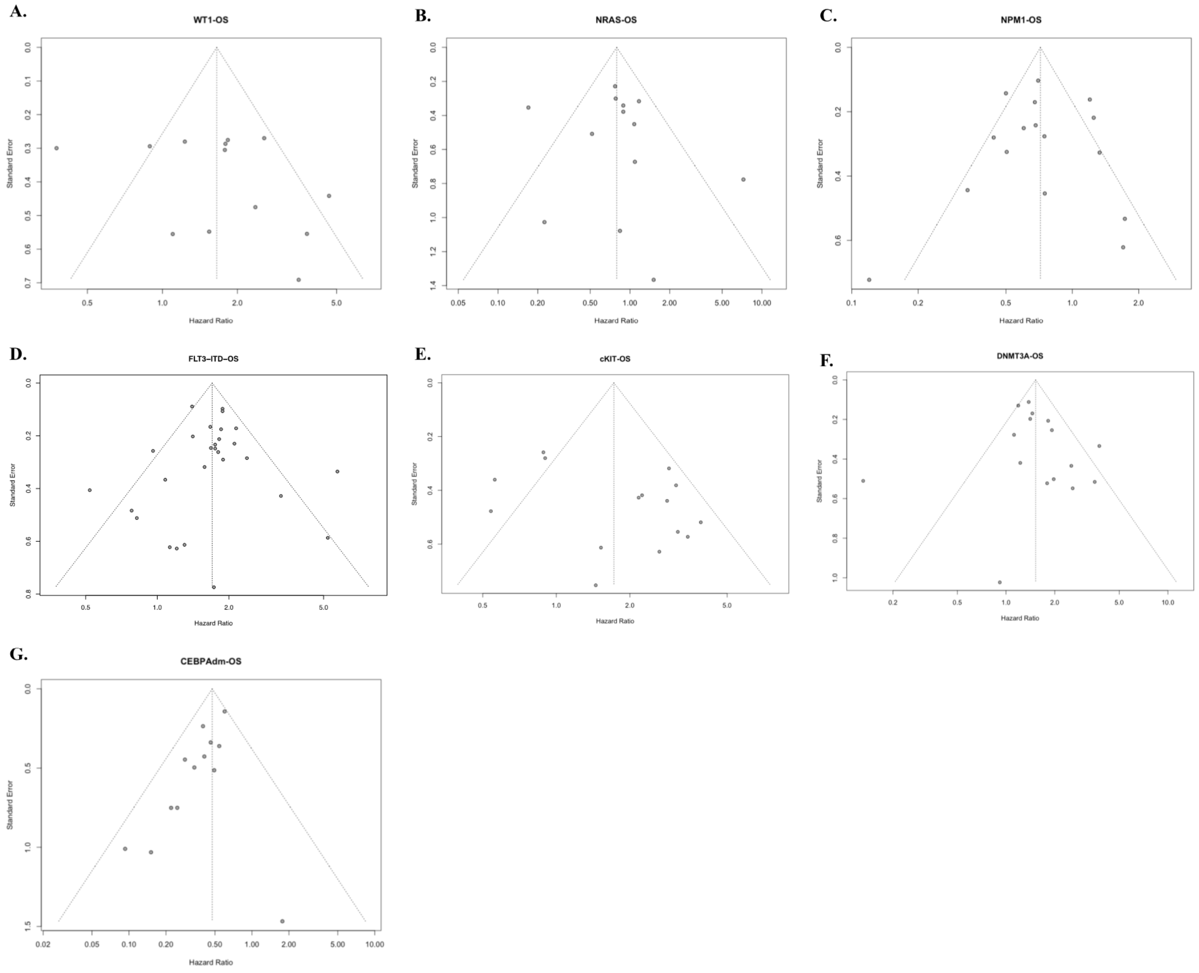

Supplementary Figure S6. Funnel plot for the publication bias test of the tested genes mutations in OS.

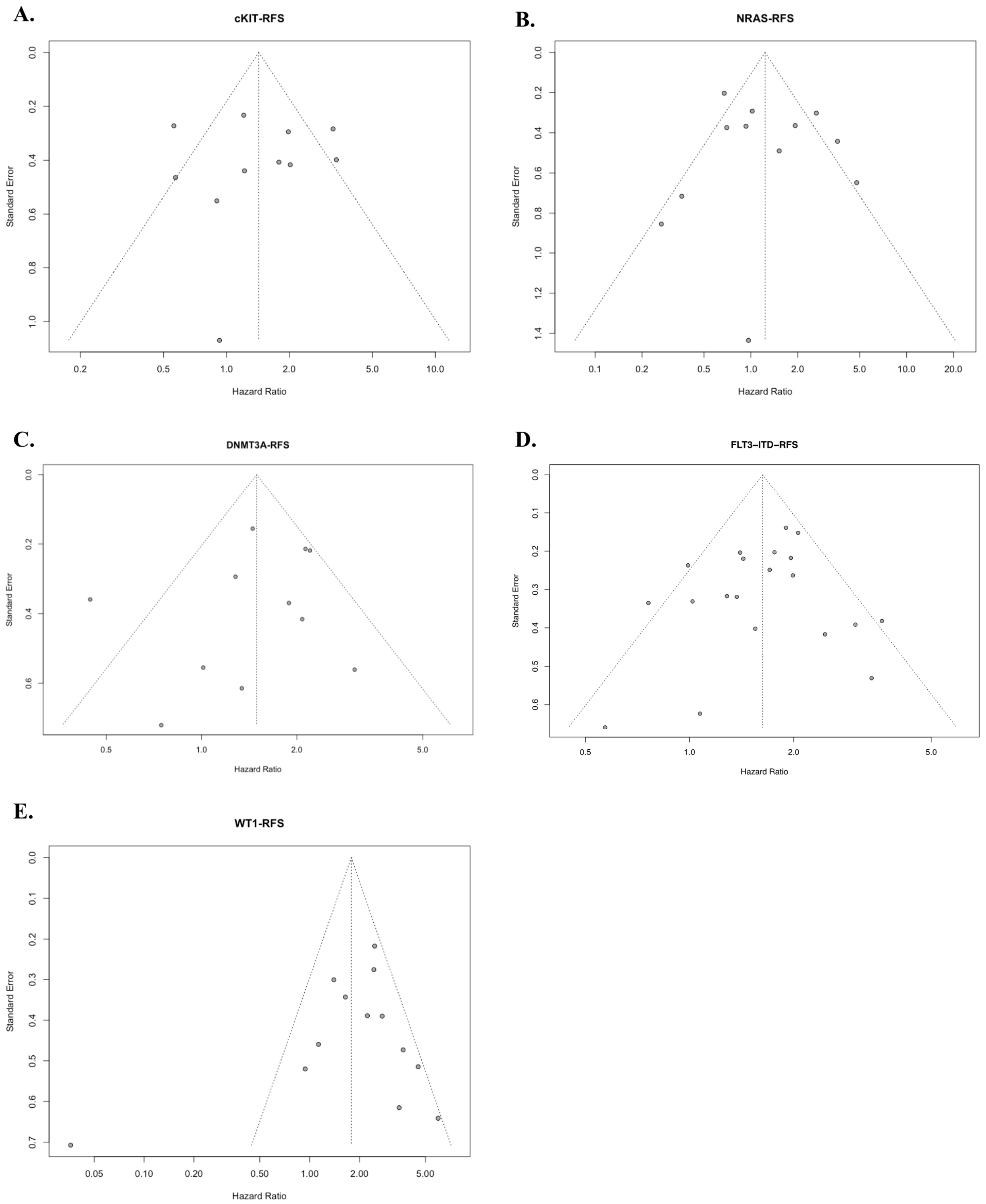

Supplementary Figure S7. Funnel plot for the publication bias test of the tested genes mutations in RFS.

Supplementary Table S1. The Prognostic impact of somatic gene mutations on over-all survival and relapse free survival of de novo AML patients.

Gene	Study ID	Study sample size	Number of carriers	OS HR (95%CI)	RFS HR (95%CI)
ASXL2	Yamato G./ 2017	369	23	0.66 (0.8-5.51)	NA
BRAF	Papaemmanuil E./ 2016	1540	9	1.4 (1.1–1.8)	NA
CCND3	Matsuo H./ 2020	160	9	1.58 (0.43-4.57)	0.80 (0.26-2.04)
CREBBP	Wei H./ 2022	171	17	0.67(0.09-5.03)	0.65. (0.20-2.09)
CUX1	Wei H./ 2022	171	9	3.17 (0.84-11.98)	2.44 (0.82-7.25)
EP300	Wei H./ 2022	171	12	0.92 (0.12-6.89)	0.92 (0.29-2.95)
ETV6	Sun Y./ 2022	74	2	15.70 (0.86-287.56)	NA
EVI1	Ribeiro A.F.T./ 2012	415	40	1.43 (0.80-2.30)	1.59 (0.80-2.80)
FAT1	Zhong WJ/ 2021	113	17	1.24 (0.63-2.46)	NA
FBXW7	Yu G./ 2018	64	5	4.13 (1.35-12.62)	2.66 (0.77-9.19)
JAK2	Christen F./ 2019	143	15	3.26 (1.64–6.48)	NA
JAK3	Wei H./ 2022	171	9	3.24 (0.95-11.07)	2.17 (0.86-5.47)
MET	Yu G./ 2018	64	8	3.44 (1.36-8.71)	2.36 (0.89-6.28)
MLH1	Yu G./ 2018	64	6	2.25 (0.77-6.58)	1.61 (0.56-4.65)
NOTCH	Han H./ 2023	878	27	1.58 (0.98-2.54)	2.15 (1.17-3.98)
NOTCH2	Wei H./ 2022	171	12	0.94 (0.13-7.04)	1.09 (0.34-3.50)
PAX5	Yu G./ 2018	64	4	1.4 (0.33-5.97)	1.15 (0.27-4.86)
PCLO	Wu L.-X./ 2021	158	28	NA	0.39 (0.12-1.28)
RAD21	Christen F./ 2019	143	18	0.39 (0.12–1.25)	0.40 (0.13-1.29)
SH2B3	Wei H./ 2022	171	9	2.14 (0.50-9.22)	0.72 (0.17-2.95)
STAG2	Matsuo H./ 2020	160	8	0.17 (0.01-0.93)	1.03 (0.27-3.26)
TERT	Aref S./ 2014	153	18	2.99 (1.30-6.60)	NA
UBTF-ITD	Kaburagi T. / 2023	369	5	5.65 (0.86-36-96)	NA
ZRSR2	Papaemmanuil E./ 2016	1540	13	1.3 (1.0–1.7)	NA

Abbreviations: OS, over-all survival; RFS, relapse free survival; HR, hazard ratio; 95%CI, 95% confidence interval; NA, not available.

Supplementary Table S2. Subgroup analyses of OS on somatic genes mutations impact in de novo AML patients.

Comparison variables	NRAS				IDH2			
	K	HR (95%CI)	I^2 (%), P_h	P*	K	HR (95%CI)	I^2 (%), P_h	P*
Total	13	0.79 (0.51-1.23)	62.2%, 0.0015	0.30	6	1.06 (0.62-1.82)	75.8%, 0.0010	0.84
**Region**								
European	2	0.95 (0.62-1.45)	0.00%	0.55	4	1.01 (0.57-1.80)	89.50%	0.86
Non-european	11	0.77 (0.45-1.31)	66.00%		2	1.18 (0.25-5.67)	72.30%	
**Age group**								
Pediatric	4	1.35 (0.58-3.15)	54.70%	0.28	0	NA	NA	0.07
Adult	6	0.56 (0.28-1.12)	64.90%		3	1.68 (0.73-3.83)	87.70%	
Mixed	3	0.75 (0.53-1.06)	0.00%		3	0.77 (0.61-0.97)	0.00%	
**Data type**								
Multivariate	9	0.63 (0.41-0.97)	59.50%	0.06	5	1.12 (0.57-2.18)	80.60%	0.48
Others*	4	1.60 (0.67-3.84)	41.90%		1	0.86 (0.64-1.16)	NA	

Abbreviations: K, number of studies; P*, Test for subgroup differences (random effects model); Others*, include univariate analysis or data from Kaplan Meier Curve.

Supplementary Table S3. Subgroup analyses of RFS on somatic genes mutations impact in de novo AML patients

Comparison variables	NPM1				WT1				NRAS			
	K	Pooled HR (95%CI)	I^2 (%), P_h	P*	K	Pooled HR (95%CI)	I^2 (%), P_h	P*	K	Pooled HR (95%CI)	I^2 (%), P_h	P*
Total	9	0.67 (0.41-1.11)	72.9%, 0.0003	0.12	13	1.78 (0.94-3.39)	74.8%, < 0.0001	0.08	12	1.23 (0.78-1.95)	69.5%, 0.0002	0.37
**Region**												
European	5	0.73 (0.39-1.37)	72.20%	0.66	5	2.10 (1.29-3.41)	43.00%	0.57	3	1.06 (0.24-4.64)	77.00%	0.79
Non-european	4	0.57 (0.23-1.43)	78.40%		8	1.51 (0.53-4.26)	82.80%		9	1.30 (0.82-2.07)	70.20%	
**Age group**												
Pediatric	1	0.52 (0.13-2.10)			3	2.04 (1.14-3.67)	36.10%		3	1.19 (0.34-4.17)	78.30%	
Adult	7	0.74 (0.39-1.38)	76.40%	0.36	6	1.47 (0.33-6.48)	87.40%	0.91	6	1.78 (0.90-3.52)	43.00%	0.06
Mixed	1	0.40 (0.23-0.69)			4	2.05 (1.42-2.97)	29.80%		3	0.72 (0.53-1.00)	0.00%	
**Data type**												
Multivariate	6	0.60 (0.29-1.23)	73.50%	0.53	8	1.22 (0.45-3.29)	82.80%	0.09	8	1.09 (0.63-1.88)	69.80%	0.49
Others	3	0.82 (0.41-1.63)	80.80%		5	3.04 (2.04-4.53)	0.00%		4	1.56 (0.65-3.77)	60.80%	

Abbreviations: K, number of studies; P*, Test for subgroup differences (random effects model); Others*, include univariate analysis or data from Kaplan Meier Curve.

Supplementary Table S4. Egger's and Begg's tests for publication bias.

		Egger's test	Begg's test
	**Endpoints**	**p value**	**p value**
DNMT3A	OS	0.6010	0.4713
	RFS	0.5127	0.3918
FLT3-ITD	OS	0.8582	0.3043
	RFS	0.4217	0.5592
cKIT	OS	0.1137	0.9605
	RFS	0.9188	0.3115
WT1	OS	0.2944	0.9029
	RFS	0.4053	0.9029
NRAS	OS	0.6462	0.9029
	RFS	0.6519	0.6808
NPM1	OS	0.9432	0.7871
CEBPAdm	OS	0.0142	0.1127
